# Longitudinal Associations of Homeownership Status with Mortality and Geriatric Outcomes in Older Americans

**DOI:** 10.1093/geroni/igaf122.394

**Published:** 2025-12-31

**Authors:** Yi Wang, Lucero Paredes, Thomas Gill, Robert Becher

**Affiliations:** Yale University, New Haven, Connecticut, United States; Yale University, New Haven, Connecticut, United States; Yale University, New Haven, Connecticut, United States; Yale University, New Haven, Connecticut, United States

## Abstract

Homeownership is a key social determinant of health, and its absence has been associated with poor self-rated health and hospitalization among older persons. However, the associations between homeownership status and subsequent mortality and incident geriatric conditions remain understudied among community-living older Americans. Using data from the 2015 cohort of the National Health and Aging Trends Study (NHATS)—a nationally representative study of Medicare beneficiaries aged 65 years or older—we evaluated whether homeownership status is associated with mortality and the incidence of frailty, disability, and dementia over a 5-year follow-up period. Mortality was determined from linked Medicare records, while the geriatric outcomes were determined using annual NHATS surveys. Homeownership status was classified as non-homeowners (renters or those in other housing arrangements) versus homeowners. We applied time-varying Cox proportional hazards models with homeownership status as a time-dependent variable, adjusting for demographic and socioeconomic factors to estimate hazard ratios (HR) and 95% confidence intervals (CI). NHATS survey weights were used to generate nationally representative estimates. Among the 7499 participants (representing 40,728,543 older Americans) in 2015, 26.0% were non-homeowners. Over the 5-year follow-up period, being a non-homeowner was significantly associated with higher mortality (HR = 1.64, 95% CI = 1.50-1.80) and higher incidence of frailty (HR = 1.39; 95% CI = 1.14-1.69), disability (HR = 1.44; 95% CI = 1.23-1.69), and dementia (HR = 1.84, 95% CI = 1.49-2.26). Overall, these findings highlight non-homeownership as an important yet underrecognized risk factor for mortality and geriatric outcomes among community-living older Americans.

